# Perspective of mesenchymal transformation in glioblastoma

**DOI:** 10.1186/s40478-021-01151-4

**Published:** 2021-03-24

**Authors:** Yona Kim, Frederick S. Varn, Sung-Hye Park, Byung Woo Yoon, Hye Ran Park, Charles Lee, Roel G. W. Verhaak, Sun Ha Paek

**Affiliations:** 1 Department of Neurosurgery, Cancer Research Institute and Ischemic/Hypoxic Disease Institute, Seoul National University College of Medicine, Seoul, 03080 Korea; 2 The Jackson Laboratory for Genomic Medicine, Farmington, CT 06032 USA; 3 Department of Neurosurgery, Cancer Center Amsterdam, Amsterdam University Medical Centers, VU University Medical Center, Amsterdam, The Netherlands; 4 Department of Pathology, Seoul National University Hospital, Seoul, 03080 Korea; 5 Division of Hemato-Oncology, Department of Internal Medicine, Seoul Paik Hospital, Seoul, 04551 Korea; 6 Department of Neurosurgery, Soonchunhyang University Seoul Hospital, Seoul, 04401 Korea

**Keywords:** Glioblastoma, Mesenchymal transition, Master transcriptional regulator, Transcriptomic plasticity, TAMs

## Abstract

Despite aggressive multimodal treatment, glioblastoma (GBM), a grade IV primary brain tumor, still portends a poor prognosis with a median overall survival of 12–16 months. The complexity of GBM treatment mainly lies in the inter- and intra-tumoral heterogeneity, which largely contributes to the treatment-refractory and recurrent nature of GBM. By paving the road towards the development of personalized medicine for GBM patients, the cancer genome atlas classification scheme of GBM into distinct transcriptional subtypes has been considered an invaluable approach to overcoming this heterogeneity. Among the identified transcriptional subtypes, the mesenchymal subtype has been found associated with more aggressive, invasive, angiogenic, hypoxic, necrotic, inflammatory, and multitherapy-resistant features than other transcriptional subtypes. Accordingly, mesenchymal GBM patients were found to exhibit worse prognosis than other subtypes when patients with high transcriptional heterogeneity were excluded. Furthermore, identification of the master mesenchymal regulators and their downstream signaling pathways has not only increased our understanding of the complex regulatory transcriptional networks of mesenchymal GBM, but also has generated a list of potent inhibitors for clinical trials. Importantly, the mesenchymal transition of GBM has been found to be tightly associated with treatment-induced phenotypic changes in recurrence. Together, these findings indicate that elucidating the governing and plastic transcriptomic natures of mesenchymal GBM is critical in order to develop novel and selective therapeutic strategies that can improve both patient care and clinical outcomes. Thus, the focus of our review will be on the recent advances in the understanding of the transcriptome of mesenchymal GBM and discuss microenvironmental, metabolic, and treatment-related factors as critical components through which the mesenchymal signature may be acquired. We also take into consideration the transcriptomic plasticity of GBM to discuss the future perspectives in employing selective therapeutic strategies against mesenchymal GBM.

## Introduction

Glioblastoma (GBM) is the most aggressive and infiltrative primary brain tumor in adults. The current standard treatment regimen for patients with newly diagnosed GBM was established in 2005, and consists of maximal safe surgical resection followed by concurrent chemoradiation with temozolomide (TMZ), an FDA-approved alkylating agent [145]. Despite this treatment, the 5-year recurrence rate of GBM after initial treatment is as high as 90% [5, 163]. Managing recurrent GBMs is a challenging problem because subsequent treatment options are limited and recurrent tumors often manifest in a more aggressive and infiltrative pattern. Thus, the development of effective and new treatment modalities for both newly diagnosed and recurrent GBMs is of exceptional importance [145].

Particularly, in response to growing interest of the personalized medicine for cancer patients, there have been many studies that divided and characterized the molecular background of GBM based on its clinical, genomic, transcriptomic, epigenomic and proteomic features. An attempt to transcriptionally categorize GBM tumors was made by Phillips et al. who identified and characterized three signatures of GBM, known as the proliferative, proneural, and mesenchymal signatures, based on gene expression profiling and subsequent longitudinal analysis of glioma cases [114]. In 2010, a group of researchers from TCGA built upon this work to identify four transcriptomic subtypes of GBM—proneural, neural, classical and mesenchymal—based on unsupervised transcriptomic analysis of 202 newly diagnosed GBM cases, which showed strong associations with genomic alterations of genes such as *TP53, EGFR* and *NF1* [155]. At this point, GBM tumors harboring the mesenchymal signature have gained much attention due to their highly aggressive natures compared to those with other transcriptomic signatures [10, 35, 89, 181, 186]. Notably, mesenchymal GBM was found to be characterized by an increased presence of immune cells, as compared to other transcriptional subtypes [62], questioning whether mesenchymal transcriptional signature is intrinsically captured in GBM cells or a byproduct of bulk RNA-sequencing being contaminated by non-neoplastic cells. Wang et al. through tumor cell-intrinsic gene expression analysis, have revealed that the TCGA-derived IDH wild-type GBM transcriptomic signatures can be reduced to the proneural, classical and mesenchymal subtypes, with the neural subtype representing normal cell contamination [161]. Furthermore, other studies using single-cell RNA-sequencing have revealed that some glioma cells themselves exhibit mesenchymal signature, suggesting that this signature is far from being simply tissue artifact [26, 177].

Although mesenchymal GBM was initially considered as the most aggressive transcriptomic signature in GBM [16], the significant survival difference was observed only when restricting the samples with low transcriptional heterogeneity, questioning the studies on the transcriptional subtypes of GBM performed without considering transcriptional heterogeneity [161]. However, the wealth of studies performed on so-called “mesenchymal” phenotypes described in the present review and also other reviews collectively suggest that mesenchymal GBM exhibits relatively more aggressive characteristics compared to other transcriptional signatures [4, 9, 38]. Other classification systems of GBM have also been reported based on other parameters (e.g., DNA methylation patterns) and shown to correlate with patient prognosis and also transcriptomic signatures [66, 81, 185]. Although these classification systems may be more robust, we will not further discuss here as the focus of the present review is on the mesenchymal signature of GBM identified through transcriptomic analysis.

Generally, two types of mesenchymal GBM have been described: microenvironment-driven and microenvironment-independent mesenchymal GBM [100, 161]. Non-neoplastic cells of microenvironmental components, especially brain-resident microglia and infiltrated monocyte-derived macrophages, have been shown closely associated with the development of mesenchymal GBM [11, 35, 48, 130, 131]. Bhat et al. reported that the mesenchymal signature is lost in patient-derived glioma sphere cultures and xenograft models despite originating from mesenchymal tumors, suggesting the necessity of human tumor microenvironmental factors in acquiring and maintaining the mesenchymal phenotypic state of GBMs [11]. Furthermore, the global and regulatory transcriptional profile of tumor-associated macrophages that drive mesenchymal phenotype in GBM has recently been identified, further highlighting the association between microenvironmental components and mesenchymal GBM [131]. On the other hand, mesenchymal transformation has also been reported to occur in a glioma cell-intrinsic manner [11, 47]. It was shown that the mesenchymal phenotype of initiating GBM was maintained in derived sphere cultures and also as xenograft models, indicating that some glioma cells are capable of sustaining the mesenchymal state independently of their microenvironment [11]. Moreover, it was reported that tumor bulk cells themselves may undergo a subtype transition to mesenchymal signature under a selective pressure of treatment, and this phenomenon was not associated with stromal enrichment via a high rate of cell death in the tumor bulk [47]. Whether microenvironment-driven or -independent, the acquisition of mesenchymal signature poses a significant clinical challenge as it exemplifies the plasticity of GBM and underlies the real problem of treatment resistance [10, 11, 24, 93, 116]. Therefore, understanding the impact of cell-intrinsic and -extrinsic cues on the intratumoral variability in GBM is critical to develop and optimize the multimodal therapeutic strategies for GBM patients. In this review, we will discuss the current molecular pathobiology in mesenchymal transformation in GBM, while focusing on the dynamics and molecular factors associated with the mesenchymal transcriptional state and also their clinical implications.

## Clinical challenges of mesenchymal glioblastoma

The development of mesenchymal GBM has been found tightly associated with that of resistance to therapeutic agents widely applied and clinically tried for GBM patients [11, 77, 115, 133, 135]. The relationship between the mesenchymal signature of GBM and radiotherapy, which is a part of the current standard-of-care for GBM treatment, has intensively been studied [11, 47]. Bhat et al. demonstrated a mechanistic link between radiation resistance and mesenchymal GBM, presenting evidence of therapeutic risk of ionizing radiation for GBM patients. In this study, they proposed a metagene score of the mesenchymal signature, which consists of proteins such as YKL40, SERPINE1, TIMP1 and TGFBI, and showed that GBM patients with higher mesenchymal metagene score are associated with poor response to radiation regardless of IDH1/2 mutational status [11]. Additionally, Holliday et al. utilized a genetically engineered mouse model of proneural glioma and revealed that the mesenchymal transition of tumor occurred within 6 h upon radiation, suggesting an intrinsic ability of GBM cells to cope with the therapeutic stress [47]. Moreover, radiation-associated mesenchymal differentiation in GBM was found to contribute to resistance to the alkylating agent TMZ as well [70].

It is now evident that tumor-associated macrophages and microglia (TAMs), which account for as many as 30–50% of the cell populations in the GBM microenvironment, are the critical stromal elements whose bidirectional communications with glioma cells are associated with the aggressiveness of GBM tumor as well as resistance to standard therapies [48, 79]. Recently, Akkari et al. who analyzed the phenotypic heterogeneity and plasticity of distinct TAM populations in the irradiated GBM microenvironment, have shown that the number of TAMs is progressively accumulated throughout the course of the 5-day fractionated radiotherapy regimen in GBM preclinical mouse models [2]. This observation is further supported by the findings of Doan et al. who showed that the global mRNA expression changes following irradiation are associated with a positive regulation of macrophage chemotaxis [31]. These results suggest that radiotherapy may elicit the recruitment of TAMs, which have also been reported to induce mesenchymal differentiation in GBM cells followed by radioresistance [11, 153].

Furthermore, resistance to antiangiogenic therapy, such as bevacizumab, which is a recombinant human monoclonal antibody acting against the vascular endothelial growth factor (VEGF), has been found closely linked to the mesenchymal phenotype of GBM [115]. Sandmann et al. in their retrospective analysis of the AVAglio (Avastin in Glioblastoma) Trial, found that only IDH1 wild-type GBM patients with proneural subtype derived both overall and progression-free survival benefit compared to placebo group [133]. Although GBM patients with mesenchymal subtype experienced longer progression-free survival upon bevacizumab treatment than placebo, their overall survival was not increased, supporting the notion that tumor progression features visible through intra-patient imaging suppressed by anti-VEGF treatment are most readily present in mesenchymal GBM.

Aforementioned remarks of mesenchymal GBM present a critical issue of the potential therapeutic risk of both current standard-of-care and novel treatment modalities. However, in order for mesenchymal transition to be established as a key target for GBM adjuvant therapy, additional studies are required to understand the transcriptome-wide architecture of intratumoral variability in GBM.

## The transcriptome of mesenchymal glioblastoma

Our understanding of the transcriptional heterogeneity was extended from intertumoral to intratumoral level when GBM tumors were analyzed at multiple spatial scales and also at single-cell level. Sottoriva et al. by analyzing spatially distinct GBM fragments, demonstrated that different transcriptomic subtypes are displayed within the same tumor [142]. Also, Patel et al. showed that established GBM transcriptional subtype classifiers are variably expressed across individual cells within a tumor. These studies demonstrate that multiple transcriptomic signatures associated with cellular states coexist within a GBM tumor, suggesting that the expression profile of bulk tumors represents the average of highly heterogeneous transcriptional state admixtures of GBM cells [108]. Indeed, intratumoral heterogeneity suggests a critical concern for GBM treatment, as the collapse of tumor cells with a certain phenotype may result in the initiation and proliferation of tumor cells with other phenotypes, which may lead to mesenchymal transformation in GBM. Therefore, understanding the intratumoral transcriptional heterogeneity and how it may affect the course of GBM progression towards the mesenchymal signature is critical.

Many studies have reported the cellular and phenotypic plasticity of GBM transcriptome not only as the main driver of intratumoral heterogeneity, but also a characteristic phenomenon during tumor evolutionary dynamics [158, 161]. In particular, the significance of transcriptomic subtype transitions between diagnosis and recurrence and in response to radio/chemotherapy has increasingly been recognized due to their contribution to the development of mesenchymal-related characteristics [38, 98, 104, 114, 135]. In the following sections, we will highlight some of the important findings regarding the transcriptome of mesenchymal GBM in relation to its transcriptional network, master transcriptional regulators, and the signaling pathways and factors that are hijacked by GBM to acquire the mesenchymal phenotype.

### The transcriptional network of mesenchymal glioblastoma

In response to increasing knowledge of the molecular characteristics of GBM, Carro et al. have drawn the first comprehensive map of the transcriptional network of mesenchymal signature of GBM through reverse-engineering and an unbiased microarray technique [16]. In this work, the authors utilized context-specific regulatory network models and identified mesenchymal gene expression signature, which generally consisted of several transcriptional factors and their regulons. Importantly, the transcriptional network of mesenchymal GBM was found to be closely intertwined with Nuclear Factor-κB (NF-κB) signaling pathway [11, 169]. NF-κB is a ubiquitous transcription factor known to play a crucial role in aggressive mesenchymal differentiation in virtually all types of malignancies, including GBM [72, 148]. Generally, NF-κB is activated by a variety of both cell-extrinsic (e.g., microenvironmental factors) and -intrinsic (e.g., genomic aberrations) factors and subsequently conducts an orchestra of transcription factors and co-regulating partners, such as STAT3 and HIF-1α, to potentiate mesenchymal program [37, 59]. In particular, NF-κB has been shown to directly induce the expression of mesenchymal proteins (e.g., CD44, N-cadherin, Vimentin) and regulate the expression of key molecules that promote inflammatory microenvironment (e.g., TNFα, CCL2, IL-6) [28, 32, 90].

One of the crucial findings from analyzing the transcriptional network of mesenchymal GBM is the identification of master transcriptional regulators, which are critical in inducing and sustaining the mesenchymal properties of GBM [9]. The molecular and clinical aspects of these master transcriptional regulators are discussed next.

### Master transcriptional regulators of mesenchymal glioblastoma

Cancer-associated master transcriptional regulators are proteins that govern and regulate transcriptional cellular state of tumor and may thus be associated with potential therapeutic vulnerabilities [139]. In many types of cancer, it has been shown that genetically and/or pharmacologically inhibiting a master transcriptional regulator and its downstream signaling pathways may be a promising therapeutic strategy [3, 12, 117, 124, 128]. An attempt to identify master regulators of mesenchymal GBM was first initiated by Carro et al. who suggested STAT3 and C/EBPb as synergistic master transcriptional regulators for mesenchymal GBM [16]. Their co-expression was associated with reprogramming of neural stem cells into an aberrant mesenchymal lineage, while their down-regulation resulted in the collapse of the mesenchymal signature and reduced aggressiveness of the tumor. Interestingly, the concurrent and synergistic activity of STAT3 and C/EBPb as master mesenchymal regulators seemed to oppose their normal biological roles—astrocytic differentiation and neurogenesis, respectively—in the developing nervous system [99, 105]. The authors hypothesized that GBM cells have an ability to tolerate such an “abnormal” situation by activating downstream signaling pathways leading to an aberrant mesenchymal transformation. In addition, although sitting at a less hierarchical position in the regulatory network of the mesenchymal transcriptome than STAT3 and C/EBPb, FOSL2 and RUNX1 were also identified as potential master regulators for mesenchymal transformation in GBM [16]. Interestingly, epigenetic changes on the promoter-associated methylation sites of these two master regulators were found to be associated with mesenchymal transition accompanied by multitherapy resistance [135].

Another master regulator was found to be *transcriptional coactivator with PDZ-binding motif* (TAZ), whose up-regulation triggered the expression of mesenchymal-related proteins and aberrant osteoblastic and chondrocytic differentiation in proneural glioma stem cells in a transcriptional enhanced associate domain (TEAD)-dependent fashion. Interestingly, inferred downstream targets of TAZ largely did not overlap with those of STAT3 and C/EBPb. Such result suggests that TAZ may be an independent modulator of the mesenchymal signature and that multiple routes leading to the mesenchymal phenotype in GBM exist [10]. More recently, hyperactivation of TAZ was found to be associated with mesenchymal transition and tumor necrosis in GBM [173]. Of note, TAZ, and its paralog *Yes-associated protein* (YAP), participate as downstream transcription coactivators in the Hippo signaling pathway, which is an evolutionarily conserved regulator of tissue growth [50]. Dysregulation of the pathway has been reported to be associated with cancer development and chemoresistance in variety types of cancer, including GBM [15, 180]. As recently reviewed by Masliantsev et al. activating large tumor suppressor kinase 1/2 (LATS1/2)-dependent inhibitory signals that phosphorylate TAZ/YAP and directly disrupting TAZ/YAP-TEAD-mediated transcription may be effective therapeutic approaches to target the Hippo pathway [88]. The therapeutic potential of the benzoporphyrin derivative verteporfin, an inhibitor of TAZ/YAP-TEAD complex, was recently examined and it was shown that verteporfin suppressed expression of TAZ/YAP transcriptional targets and induced apoptosis of EGFR-amplified/mutant GBM cells [156]. As EGFR amplification is a prominent characteristic in classical subtype of GBM [155], it would be relevant to examine the efficacy of verteporfin in inhibiting mesenchymal transition in GBM by targeting TAZ. Moreover, it was found that NF-κB controls the expression of these three master transcription factors to potentiate mesenchymal differentiation in proneural glioma sphere cultures, which subsequently exhibit an enrichment of CD44^+^ subpopulations and radioresistant phenotypes [11, 169].

Furthermore, the interplay between microenvironmental factors and the activity of master transcriptional regulators has been implicated. Generally, necrosis and hypoxia are considered as crucial pathobiological features of the neoplastic microenvironment [49]. Interestingly, it has been observed that the expression of master mesenchymal regulators, especially C/EBPb and STAT3, is significantly associated with the development of necrotic and hypoxic microenvironment of GBM [9, 25]. As mentioned previously, macrophages and microglia are the integral components of the tumor microenvironment, creating a supportive stroma for GBM cell expansion and invasion [119]. Although commonly referred to as “TAMs” as a collective term, these are indeed two separate cellular entities with different ontogeny [48], and their ratio has recently been demonstrated to vary depending on the stage of GBM progression [2]. Interestingly, each of these cellular entities has been reported to modulate the activity of the master transcriptional regulator, especially STAT3, suggesting that they have differential, and yet, common roles in promoting mesenchymal transition in GBM. Dumas et al. reported that mTOR-dependent regulation of STAT3 and NF-kB activity in microglia were induced by GBM-initiating cells, which subsequently promote an immunosuppressive microglial phenotype, and that such mTOR activity is most significantly correlated with tumor-associated microglia signatures in the mesenchymal subgroup of GBM [33]. Moreover, the interaction between GBM-released factors and monocytes, which are the precursor to macrophages, has also been identified to contribute to the immunosuppressive microenvironment of mesenchymal GBM [32]. It has been shown that monocytes preferentially take up GBM-derived exosomes, which traverse the monocyte cytoplasm and mainly release STAT3, thereby triggering up-regulation of programmed death ligand 1 and skewing monocytes toward the immune suppressive M2 phenotype [40].

It is important to note that not all mesenchymal GBMs are regulated by these identified master transcriptional regulators, implying that there are still undiscovered master regulators that may regulate the mesenchymal properties of GBMs working synergistically or even independently of the previously identified master regulators. Intriguingly, studies on the master regulators of mesenchymal GBM raise a critical question as to whether these regulators are the master transcription factors of the mesenchymal state of glioma cells or those of the tumor microenvironmental components or those of both. Nonetheless, identification of additional novel master regulators may provide a clue as to how to evade mesenchymal differentiation-associated therapeutic risk and subsequently aid in the development of effective therapeutic intervention against high-grade glioma.

### Transcriptomic plasticity

It is now a well-established notion that GBM is a dynamic neoplasm whose transcriptome is capable of undergoing transition in response to selective pressure in different biological and pathophysiological settings. Such plasticity accounts for not only a high degree of inter- and intratumoral transcriptional heterogeneity, but also a birth of multitherapy resistant clones upon recurrence and/or in response to treatments.

It has been reported that proneural GBMs may acquire therapeutic resistance and more aggressive, angiogenic and hypoxic potential by shifting their transcriptomic and phenotypic signatures toward mesenchymal GBMs [11, 38, 91]. Such transcriptomic plasticity of GBM upon treatment and/or recurrence is often referred to as a “proneural-to-mesenchymal transition”, or PMT [9]. This phenomenon is generally described analogous to epithelial-to-mesenchymal transition (EMT), which is one of the dominant features driving invasiveness and metastasis in carcinomas [144]. An increasing amount of evidence has demonstrated the existence of PMT and suggested that first-line therapy for primary disease may not effectively work for recurrent tumor due to this process [45, 47, 70]. However, recurrent GBMs, especially those that have undergone mesenchymal transformation, have been reported to be associated with an increased presence of TAMs overall [79, 161], raising an issue mentioned earlier regarding bulk RNA sequencing. Moreover, although PMT was initially described as a “frequent” event and post-therapeutic characteristic of GBM [114, 166], a longitudinal transcriptome analysis performed by Wang et al. showed that 55% of tumors from IDH-wildtype GBM patients retained their original transcriptional subtype at recurrence and also that the frequency of PMT was not significantly higher than that of other subtype transitions [161].

However, consistent observations that GBM tumors that have undergone mesenchymal transformation are associated with increased aggressiveness and multitherapy resistance highlight the importance of investigating the underlying mechanisms and associated therapeutic targets [161, 166]. Generally, molecular features, intratumoral heterogeneity, immunogenicity, microenvironmental factors, and treatments have been reported to shift GBM transcriptome towards the mesenchymal signature. In the following sections, some of the main mechanisms and factors exploited by GBM to acquire the mesenchymal phenotype are discussed.

#### Microenvironmental factors

**Fig. 1:**
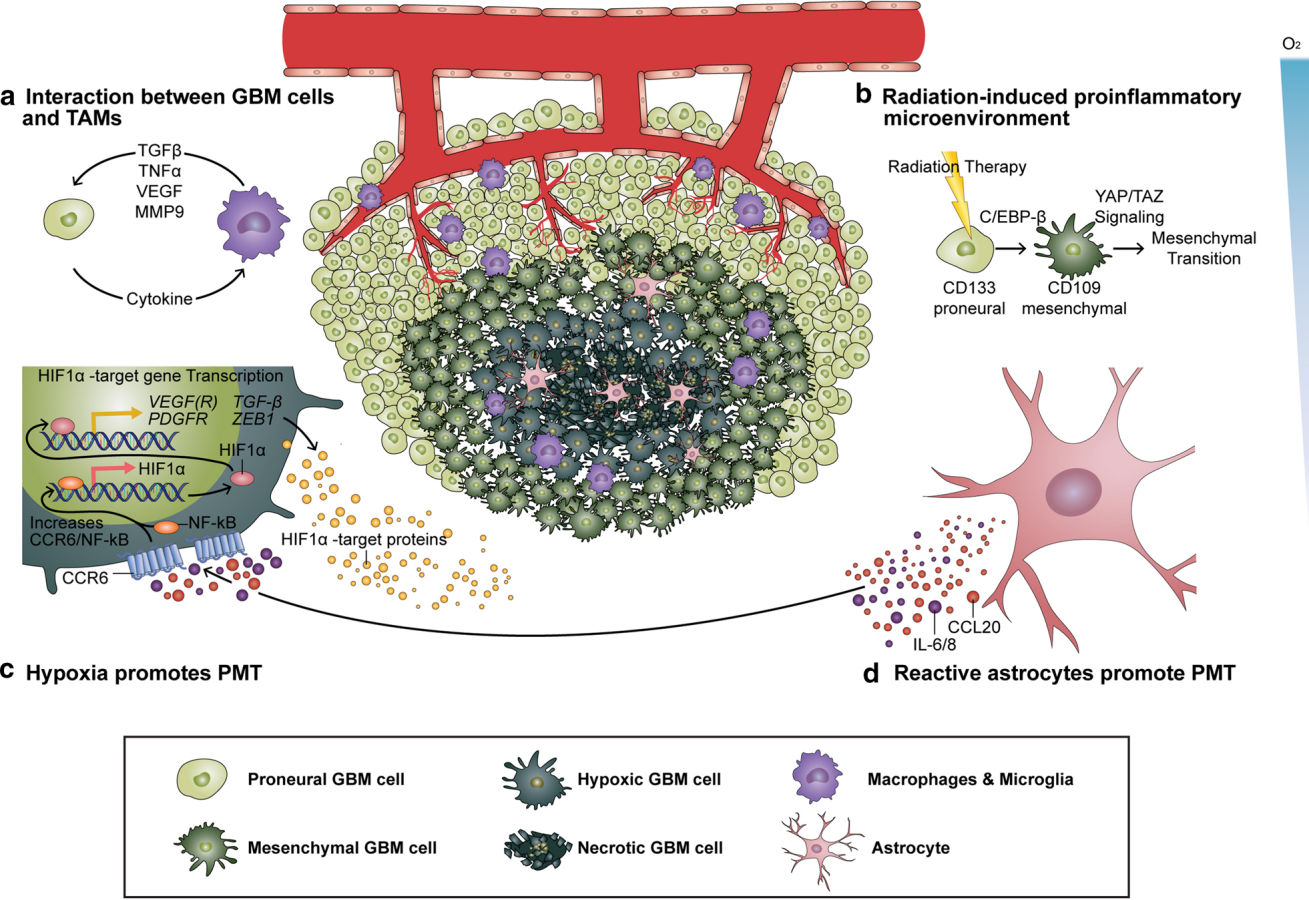
The impact of microenvironmental factors on shaping GBM transcriptome. Mesenchymal transition may be induced by a variety of microenvironmental factors such as **a** interaction between GBM cells and tumor-associated macrophages/microglia **b** proinflammatory processes induced by radiation **c** hypoxia and **d** astrocytes. As depicted in the figure, GBM is a highly heterogeneous tumor consisting of several cellular entities, such as fibroblasts, immune cells, and astrocytes and also of GBM cells harboring different transcriptomic signatures. For the representation of GBM cells with the distinctive transcriptomic states, only mesenchymal and proneural cells are portrayed

The extensive heterogeneous milieu of GBM tumors is characterized not only by several distinctive cellular entities, but also by the presence of multiple subclonal populations of GBM cells harboring different cellular states in the same tumor [100]. Importantly, dynamics associated with phenotypic heterogeneity have been reported to be instructed by the tumor microenvironment in a nonhierarchical and reversible manner [30]. Such immense heterogeneity and plasticity of GBM is now considered as a major contributing factor for overcoming selective pressures both during tumor progression and adaptation to therapeutic stresses. The coexistence of subpopulation of GBM cells of multiple transcriptional states and also that of different types of cells in the tumor microenvironment are depicted in Fig. 1 with an emphasis on their roles on promoting the mesenchymal signature.

Transcriptomic plasticity of GBM is significantly influenced by cellular heterogeneity in the tumor microenvironment [113, 134]. The continuous crosstalk between tumoral and non-tumoral cells is basically viewed as responsible for nearly all events that facilitate the self-sustained growth and invasion of neoplastic cells and therapy-resistance [159, 183]. An intimate link between the expression of a variety of immune-related genes and mesenchymal GBM reported in many studies has suggested that immunological and inflammatory processes may foster the establishment of the mesenchymal signature [11, 32, 130]. Accordingly, it has been found that the mesenchymal phenotype can be shaped by a variety of infiltrative immune cells [32, 178]. TAMs, being the largest stromal population in GBM, are generally described as integral microenvironmental components contributing to mesenchymal transition by expressing pro- (M1) (e.g., TNFα) and anti-inflammatory (M2) (e.g., TGFβ), pro-angiogenic (e.g., VEGF), and extracellular matrix remodeling factors (e.g., MMP9) (Fig. 1a) [11, 35, 119]. Also, TAMs have been shown to release a family of cytokines, which may mediate mesenchymal differentiation in an NF-κB-dependent manner. Likewise, many studies reported that mesenchymal GBM exhibits a high degree of macrophages/microglia infiltration and also necrosis compared to other transcriptional subclasses [25, 114, 155].

Heterogeneous transcriptional state of GBM cells in the tumor microenvironment can also be explained in the context of anatomical heterogeneity, which consists of various histological features [121]. Puchalski et al. utilized laser microdissection to isolate RNA from these regions and demonstrated that many of the critical mesenchymal-related signatures (e.g., HIF-1α network, TNFα signaling pathway, cell migration, and immune response) are enriched in perinecrotic/pseudopalisading zones. Similarly, necrosis was found to impact the transcriptional class of GBM in a way that non-mesenchymal signatures became more molecularly similar to the mesenchymal class with increasing levels of necrosis [11, 25]. However, more single-cell-based research is needed to determine whether glioma cells in perinecrotic/pseudopalisading areas intrinsically exhibit mesenchymal signature as these anatomical zones have been reported to harbor more TAMs than other parts of the tumor [9, 67].

Radiation-induced vascular permeability is also known to play a critical role in the process of mesenchymal transition as it has been found to result in infiltration of immune cells into the brain parenchyma and subsequently create highly immunologically active milieu through which macrophages/microglia contribute to PMT [11, 35]. Furthermore, Minata et al. have recently found that GBM cells express CD109 protein via C/EBPb in response to the radiation-induced proinflammatory microenvironment and that CD109 drives oncogenic signaling through TAZ/YAP cascade which subsequently results in PMT (Fig. 1b) [91]. Such work has revealed that the interplay between the microenvironment and master mesenchymal regulators may further enhance the plasticity of GBM transcriptome.

Another critical feature of GBM microenvironment is hypoxia, which is a key driver of both tumor growth and angiogenesis [29, 49]. Hypoxic cells have been shown to activate pro-angiogenic factors, including VEGF/VEGFR, TGFβ, and PDGFR through the stabilization of HIF-1/2α, and subsequent HIF-induced transcriptional changes elicit the recruitment of inflammatory cells, and of particular importance, the PMT (Fig. 1c) [29, 92]. For example, Joseph et al. have demonstrated that hypoxia enhances the invasive capacity of GBM cells by promoting HIF1α-ZEB1 axis-mediated mesenchymal transition [61]. Of note, chronic anti-angiogenic therapy was found to lead to excessive pruning of tumor vessels potentiating hypoxia, which, in turn, exacerbates inflammatory and angiogenic microenvironment and subsequently promotes PMT [92, 115]. These results may partly explain the treatment failure of anti-angiogenic therapy in GBM patients [43]. From these observations, it is likely that each tumor microenvironmental factor cooperates with each other to form a vicious circle of interactions through which mesenchymal transition is promoted.

Additionally, astrocytes, which co-exist with GBM cells in the hypoxic microenvironment, were found to release IL-6, -8 and CCL20, which up-regulate HIF-1α in a CCR6/NF-κB signaling-dependent manner and thereby helping GBM cells better adapt to hypoxia (Fig. 1d) [59]. The possible involvement of astrocytes in contributing to mesenchymal transition is further corroborated by a recent study by Niklasson et al. who found that the signature of mesenchymal GBM recapitulates the reactive astrocyte cell state [101].

#### Metabolic factors

Genomic abnormalities in genes encoding critical metabolic enzymes have long been recognized to be associated with pathogenesis [94, 171]. Altered energy metabolism may also impact the transcriptomic signature of GBM, which is a rapidly growing tumor with high proliferation index and neighboring geographic necrosis [151].

**Fig. 2:**
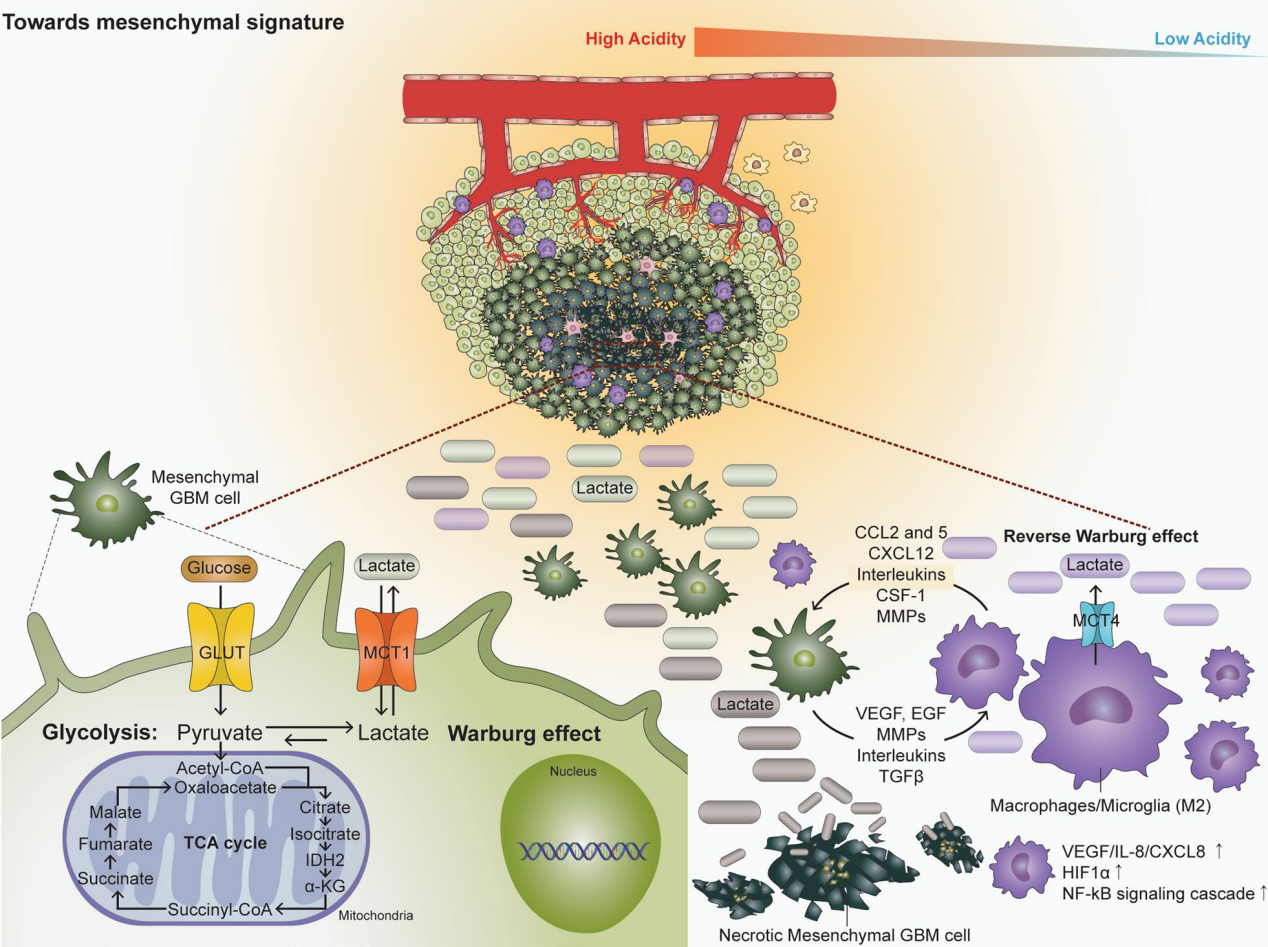
The impact of metabolic factors on shaping GBM transcriptome. Metabolic alterations associated with the mesenchymal signature of GBM. The three possible sources of lactate in GBM tumor are mesenchymal GBM cells, necrotic GBM cells, and macrophages/microglia present in the microenvironment of the tumor. Compared to GBMs of other transcriptional subtypes, mesenchymal GBM is characterized by a high production rate of lactate, which intensifies the acidity of the tumor microenvironment. Furthermore, the interaction between GBM cells and the attracted macrophages/microglia further promotes the mesenchymal property of the tumor

Especially, as the Warburg effect states, glucose metabolism in neoplastic cells is primarily characterized by increased glucose uptake and enhanced aerobic glycolysis, converting glucose into pyruvate which eventually results in increased production of lactate [41]. Not surprisingly, it has been reported that glycolytic activity was significantly increased in mesenchymal GBM relative to proneural GBM, and the highly glycolytic nature of mesenchymal GBM may indicate its propensity to metabolize glucose to lactic acid at an elevated rate (Fig. 2) [1, 23, 86]. Subsequently, when lactate is exported into the extracellular space through monocarboxylate transporters (MCTs), the tumor environment becomes acidic, which results in a local inflammatory response consisting of various immune cells, including TAMs. These cells, in turn, secrete cytokines and growth factors that promote mesenchymal-related characteristics in GBM cells [44, 107, 138]. It is also suggested that lactate promotes an immune-permissive microenvironment partly by stimulating the polarization of resident macrophages to the M2 state [22, 127]. Additionally, lactate has been reported to stabilize HIF-1α, activate NF-κB signaling cascade, and also induce secretion of VEGF from tumor-associated stromal cells, all of which are the characteristics of mesenchymal GBM [112, 141, 154, 155]. Interestingly, according to the reverse Warburg effect, these immune cells of recruited TAMs may also up-regulate MCT4 in an HIF-1α/NF-κB dependent manner, resulting in increased synthesis and export of lactate that may exacerbate the acidity of the tumor microenvironment and further enhance the glycolytic capacity of GBM cells [111, 147, 165]. Notably, it has been reported that mesenchymal GBM exhibits a high degree of necrosis compared to non-mesenchymal subtypes and that lactate accumulation is known to often occur within areas of necrosis, suggesting that necrotic mesenchymal GBM cells may also be an additional source of lactate in the tumor microenvironment (Fig. 2) [65].

The potential relevance of lactate in promoting the mesenchymal signature was further supported by Heiland et al. who investigated the landscape of metabolomics–transcriptomic alterations in GBM [51]. They showed that metabolites such as choline and lactate are closely associated with immune- and hypoxia-related clusters, both of which show strong enrichment in the mesenchymal signature of GBM [51]. Although a more precise and mechanistic role of dysregulated metabolism in promoting mesenchymal GBM still remains to be elucidated, these studies imply that certain metabolic features may foster the mesenchymal features as tumor progresses in an angiogenic and hypoxic tumor microenvironment [146].

#### Treatment-related factors

**Fig. 3:**
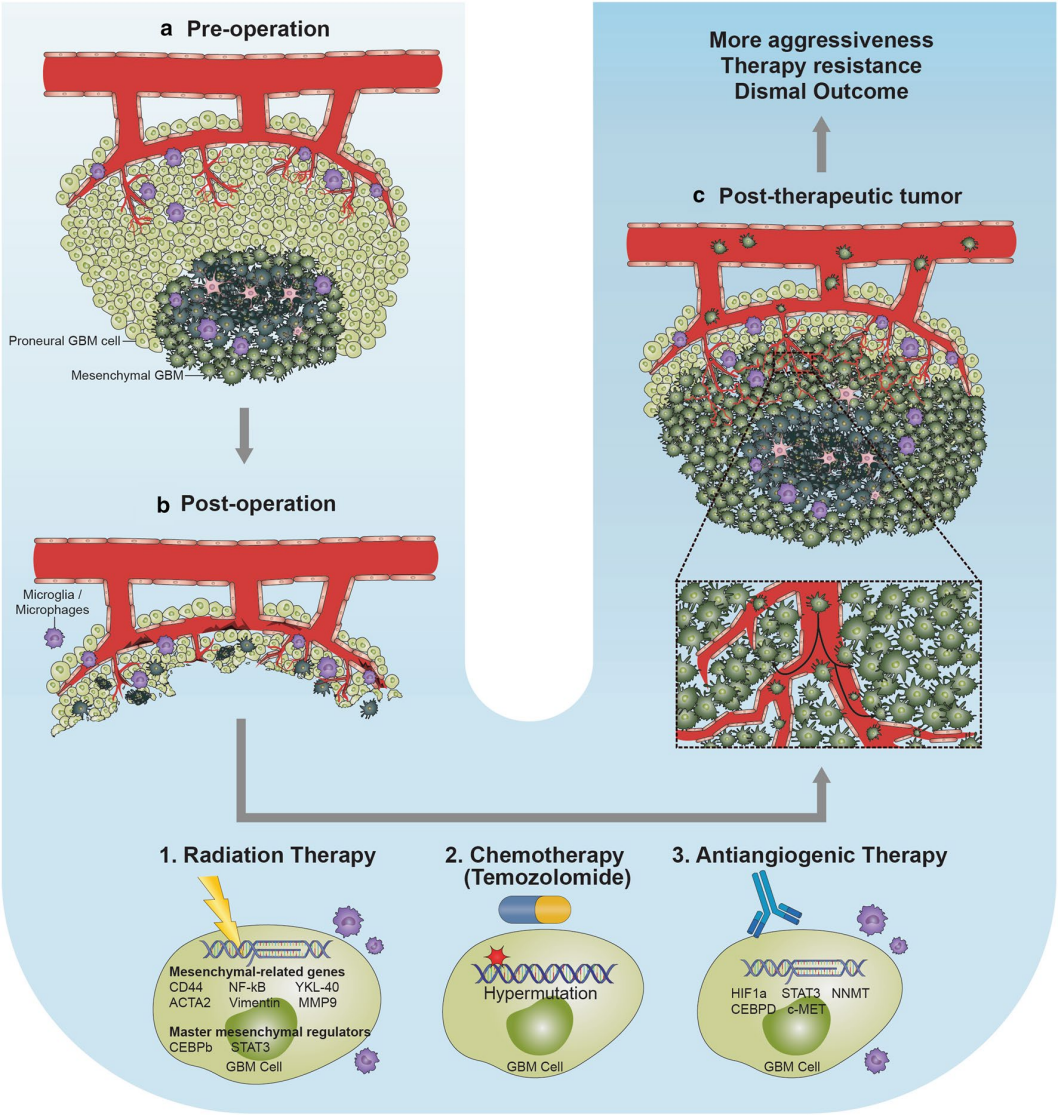
Treatment-induced proneural-to-mesenchymal transition. **a** Pre-operative tumor of proneural subtype. The tumor consists of more proneural GBM cells than mesenchymal cells. Also, the necrotic core of the tumor is shown containing hypoxic and necrotic GBM cells along with astrocytes and macrophages/microglia. **b** Post-operative state of the tumor region. Residual tumor and stromal cells remain beyond the margins after surgical resection. Such residual cells would then experience therapeutic stresses from various kinds of treatments, including radiation, chemotherapy and antiangiogenic therapy, and may be associated with treatment-induced mesenchymal transformation of the tumor. **c** Post-therapeutic tumor of mesenchymal subtype. The tumor contains more mesenchymal GBM cells than proneural cells. The recurrent tumor is extensively vascularized than the primary tumor, as newly formed blood vessels are highly branched

Although the current standard treatment resulted in the improvement of median overall survival of GBM patients from 12.1 to 14.6 months, the disease often progresses within 7–10 months, with the 2-year survival rate less than 20% [103, 176]. Such treatment failures and the high rate of recurrence of GBM have now been partly attributed to treatment-induced phenotypic and genomic changes in the recurrent tumors, and/or PMT. A typical pattern of tumor re-growth from proneural signature towards mesenchymal phenotype after surgical resection followed by various treatments is illustrated in Fig. 3.

Firstly, the therapeutic failure may be attributed to radiation therapy, which may ultimately induce mesenchymal transition in tumor as reported in many studies [9, 11, 47, 70, 91, 166, 184]. Mechanistically, radiation-induced mesenchymal transition is accompanied by the upregulation of CD44 and activation of NF-κB pathways. Particularly, radiation treatment triggered the expression of master mesenchymal regulators, such as C/EBPb and STAT3, and also mesenchymal proteins, including YKL-40, COL1A1, ACTA2, Vimentin, and MMP9, many of which are also the key players in EMT [47, 70]. Importantly, these radiotherapy-associated changes have been found to contribute to the poor response to post-radiation treatment and subsequently dismal outcome of the patients, implying a vicious circle of the radiation and the radiation-associated aftereffects [11]. Cellularly, radiation-induced transformation was associated with increased cellular motility and invasion through the expression of TGF-β, VEGF, and epidermal growth factor, whose blockade enhanced the radiation response [150, 187]. Radiation-induced mesenchymal transition has also been observed in other types of malignancy, such as colorectal cancer [64].

Based on these observations, many studies have been conducted to identify therapeutics that may prevent post-radiation side effects. Adhesion G-protein-coupled receptor, or GPR56/ADGRG1, has been found to inhibit mesenchymal differentiation and the associated radioresistance by targeting the NF-κB signaling pathway [93]. Also, YM155, a purported radiosensitizer, has been shown to prevent radiation-induced invasion in GBM by targeting STAT3 [184]. Furthermore, STAT3 blockade such as JAK2 inhibitors (AZD1480 or ruxolitinib) was found to augment the therapeutic efficacy of radiation and subsequently abrogate the mesenchymal signature in GBM [70].

Another type of therapy found to be linked to mesenchymal transformation in GBM is anti-angiogenic therapy. Due to a prominent neovascularity of GBM, anti-angiogenic therapy, such as bevacizumab and/or sunitinib, was considered hopeful in treating GBM tumors of both newly diagnosed and recurrent. However, in a large prospective phase III trial, the use of adjuvant bevacizumab resulted only in the improvement of progression-free survival from 1.5 to 4.2 months but not in the overall survival [19, 43, 73, 164]. Along with the glioma stem cell accumulation, the failure of anti-angiogenic therapy has largely been attributed to mesenchymal transformation in GBM cells [77, 115, 116]. Piao et al. have demonstrated that predominant biological processes occurring after the antiangiogenic therapy are the upregulation of genes involved in mesenchymal-related pathways, cellular migration and invasion, and also the influx of immune cells secreting chemokines and cytokines, which may act in an autocrine or paracrine fashion to potentiate mesenchymal shift in glioma cells [27, 96, 115]. Furthermore, bevacizumab has been reported to increase the uptake of glucose and its conversion into lactate, which may increase the acidity of the tumor microenvironment [36].

Additionally, whether TMZ-associated therapeutic effect, such as TMZ-induced mutagenesis, might partly and/or indirectly contribute to mesenchymal transition is worthy of further investigation [7, 60]. TMZ-induced hypermutated GBMs at recurrence was found to be associated with an increased frequency of CD8^+^ lymphocytes [161], which were also found to be present at a higher density in mesenchymal GBM than GBMs of other transcriptional subtypes [120].

Furthermore, although extracranial metastasis of GBM is extremely rare, occurring in less than 2% of the patients, GBMs have been reported to metastasize to other organs such as lungs and soft tissue of the posterior neck at the time of recurrence [126]. Based on the characteristics of mesenchymal GBM cells, it is likely that these cells may possess greater ability to metastasize extracranially, as their invasive and migratory capacity is highly increased.

All these studies collectively indicate that PMT, or mesenchymal transition in general, is a characteristic phenomenon of GBM cells in response to treatments. However, it is again important to note that 55% of tumors from IDH-wildtype GBM patients retained their original transcriptional subtype at recurrence [161], raising controversy over preclinical studies mentioned herein or implying a reversible phenotypic shift of non-mesenchymal-to-mesenchymal-to-non-mesenchymal transition. Therefore, more research is needed to reduce the discrepancy between the results obtained from preclinical and large-scale analysis of clinical datasets. Such research will be key to maximize the potential of bench-to-bedside translation for efficient adjuvant GBM therapy.

#### Therapeutic strategies against transcriptomic plasticity

The aforementioned characteristics of transcriptomic plasticity raise several critical clinical implications for GBM therapy. It is now evident that standard-of-care first-line treatment is insufficient to effectively abolish GBM whose transcriptome is capable of evolving into a distinct and more aggressive phenotype. The apparent transcriptomic plasticity of GBM suggests that the treatment regimen for GBM should also consider the oncogenic pathways that may distinctively be activated as tumor progresses and also that targeted therapy should be applied concurrently or as a part of multimodal treatment strategy.

To address this issue, several novel therapeutic modalities have been developed and intensively studied [14, 46, 56, 85]. Especially, specific inhibitors of the mesenchymal phenotype in combination with other therapeutic regimen have been suggested to synergistically inhibit GBM progression and/or mesenchymal transition [10, 11, 70]. Nevertheless, multidrug therapies could be associated with treatment complications as it was reported by Batchelor et al. who showed that the combination of TMZ with bevacizumab resulted in high toxicity and intracranial hemorrhage in a group of patients [8].

As previously discussed, TAMs are the major tumor microenvironmental cell types that play a critical role in the process of mesenchymal transformation in GBM, and therefore, targeting TAMs may be a viable therapeutic approach to not only augment the therapeutic efficacy of radiotherapy [2], but also impede the subsequent mesenchymal transition. The translational potential of targeting TAMs was investigated by Pyonteck et al. who used BLZ945, which is a brain-penetrant inhibitor of colony stimulating factor-1 receptor (CSF-1R), to target TAMs and demonstrated a significant increased survival in the treated mice [122]. An interesting observation from this study was that CSF-1R blockade did not affect the number of TAMs, but rather, decreased pro-tumorigenic M2 markers in TAMs, suggesting that TAMs are “re-educated” to perform antitumor activity. Furthermore, it was shown that CSF-1R inhibition by PLX3397, which hampers the tyrosine kinase activity of CSF-1R, prevented radiation-recruited monocytes from differentiating into immunosuppressive and pro-tumorigenic M2 macrophages [131, 143, 170]. Of note, GBMs may also develop resistance to sustained CSF-1R blockade. It was observed that > 50% of tumors relapsed after BLZ945 treatment. However, combined treatment of BLZ945 with blockades for either insulin-like growth factor-1 receptor (IGF-1R) or phosphatidylinositol 3-kinase (PI3K), the activities of which were found to be elevated in recurrent GBM, resolved the resistance to CSF-1R inhibitor [123]. Similarly, PLX3397 alone was not found effective in treating recurrent GBMs in a phase II clinical trial, suggesting that a combined therapy may be needed to augment the potential efficacy of targeting CSF-1R in GBM [13].

Unfortunately, to our knowledge, there has been no study investigating the therapeutic efficacy of CSF-1R inhibition specifically on mesenchymal GBM or whether it can prevent subtype transitioning into mesenchymal signature. The genetically engineered mouse models of glioma used by Akkari et al. to examine the combined therapeutic efficacy of CSF-1R inhibitor and radiotherapy were generated in an *Ink4a/Arf*-deficient background (platelet-derived growth factor-driven Ink4a/Arf KO), which was previously reported to closely resemble the proneural subtype of GBM [2, 47]. Given a tight association between mesenchymal GBM and the abundance of infiltrated TAMs, it would be worth investigating if mesenchymal GBM would represent the most sensitive subtype to CSF-1R inhibition compared to other transcriptional subtypes [11, 131, 137]. Of note, the two recurrent GBM patients who had best progression-free survival after PLX3397 treatment in a phase II clinical trial were found to exhibit the mesenchymal signatures [13]. Additionally, since TAMs have also been reported to be associated with angiogenesis, vasculogenic mimicry and revascularization after radiation in GBM xenografts, it will also be instructive to examine the synergistic effect of CSF-1R inhibitors with anti-angiogenic therapy, which was shown effective in high-grade serous ovarian cancer [80, 129, 162].

Oncolytic virotherapy may also circumvent mesenchymal transition in GBM by promoting the polarization state of TAMs as pro-inflammatory and antitumoral M1 phenotype. As it was discussed in the recent review by Zhang and Liu, genetically modified oncolytic viruses expressing immunomodulatory transgenes have been considered as a promising therapeutic tool for glioma treatment [182]. Combined treatment of oncolytic herpes simplex virus expressing IL-12 with two checkpoint inhibitors (anti-CTLA-4 and anti-PD-1) resulted in the regression of GBM tumor in a preclinical mouse model and, interestingly, this treatment was found to be associated with influx of macrophages of M1-like polarization state [132]. Moreover, the effectiveness of the oncolytic adenovirus Delta24-RGD was investigated which produced a prolonged M2 to M1 TAMs phenotype shift in GBM, suggesting that oncolytic virotherapy may be applied to modulate radioresistance [152, 161]. Notably, the synergistic effect of oncolytic virotherapy with radiotherapy or TMZ was also reported, raising a feasibility that oncolytic virus may effectively be used with standard-of-care treatment [6, 42, 75]. Further studies are needed to determine whether oncolytic virotherapy prevents subtype plasticity and/or mesenchymal transformation in GBM.

Genomic aberrations in neurofibromin 1 (NF1) gene have been reported as one of the major characteristics of the mesenchymal GBM, and *NF1* deficiency has been shown to recruit TAMs to the tumor site, indicating that reconstituting functional *NF1* may prevent mesenchymal transformation in GBM [155, 161]. As reviewed by Leier et al. biotechnology-based therapeutic strategies, such as cDNA replacement, CRISPR-based DNA repair and exon skipping, are being developed as a form of mutation-directed therapies to repair the *NF1* gene [71]. Furthermore, since the loss of *NF1* in glial cells has been found to be associated with increased RAS activity, targeting the RAS-downstream signaling pathways (e.g., the RAF-MEK-ERK signaling cascade) through MEK inhibition may be another viable therapeutic strategy to prevent NF1-associated mesenchymal transformation in GBM [17, 57, 78].

Currently, salvage therapy for the recurrent GBMs generally includes reoperation, fractionated re-irradiation, re-chemotherapy, gamma knife radiosurgery (GKRS) and various kinds of targeted therapies, which are usually applied to patients in clinical trials [95]. Among these, GKRS has been placed as one of the attractive and relatively safe salvage treatments for the recurrent GBMs [39, 69]. Dual treatment of radiosurgery and bevacizumab was reported to benefit both the overall (11.2–17.9 months) and progression-free survival (3.9–14.9 months) of GBM patients [95, 106]. This indicates that the combination of salvage GKRS and adjuvant chemotherapy may offer a novel treatment option to improve the prognosis of patients with recurrent mesenchymal GBMs.

Nonetheless, tumors may also progress after GKRS and it has been reported that the patterns of recurrence are similar to those of conventional radiation, implying the possible development of the mesenchymal signature in GKRS-induced response in tumor [106]. Importantly, symptomatic radionecrosis, which commonly results from avascularized tissue at the site of the GKRS target, is a well-recognized treatment risk of stereotactic radiosurgery [109]. Telangiectasis was observed to be the most prominent vasculature in the radionecrotic and/or perinecrotic region where the abundant expression of VEGF was concomitantly observed, and the reactive astrocytes, which were intensively distributed in this area, were found to be a major source of VEGF production [102]. Similarly, Yoritsune et al. reported that the perinecrotic area formed after intensive radiotherapy is mainly infiltrated by two distinct cell populations—reactive astrocytes and microglias, which were found to express VEGF and HIF-1α, respectively [175]. These studies imply that GKRS-induced symptomatic radionecrosis may be an indicator of the development of mesenchymal GBM. Although GKRS-associated transcriptomic changes have not been reported to date, it may be speculated that focal radiation may strongly activate the master mesenchymal regulators, suggesting that the therapeutic intervention of mesenchymal inhibitors may be needed to circumvent GKRS-associated complications.

Thermotherapy has also been described as a promising therapeutic modality for both newly diagnosed and recurrent GBMs and a growing body of evidence implies that it may be used as a salvage treatment option for the therapy-resistant mesenchymal GBMs. Laser interstitial thermal therapy (LITT) is a minimally invasive thermal ablation approach that surgically addresses not only symptomatic radionecrosis, but also high-grade gliomas that are treatment refractory and/or unresectable [52, 53, 136]. LITT has been reported to promote the disruption of the blood–brain barrier, thereby enhancing the effects of adjuvant chemotherapies [52, 136], suggesting that dual treatment of LITT and mesenchymal inhibitors may be useful in treating recurrent mesenchymal GBMs [54]. Another type of thermotherapy is magnetic hyperthermia, which generates heat by magnetic nanoparticles in response to the application of an external alternating magnetic field [83, 87]. Alongside producing a localized thermo-ablative effect, the therapeutic potential of magnetic hyperthermia has been described based on its synergistic effect as a chemoradiosensitizer [63, 84], and also immunomodulatory effect [140]. These suggest that magnetic hyperthermia may be applied to modulate the immunosuppressive microenvironment of mesenchymal GBM patients. Of note, such heat-based therapies have been associated with elevated expression of the family of heat shock proteins, which may reduce the efficacy of subsequent thermotherapies [34]. It has been reported that thermotolerance may be achieved by the expression of HSP90, HSP70 and HSP27, which have also been suggested to promote mesenchymal transformation in GBM [125], indicating that mesenchymal inhibitors may be needed to prevent possible thermotherapy-associated transcriptomic shift.

The synergistic and possible adverse effects of the combined therapeutic modalities, including those described here, must be actively investigated to optimize the therapeutic design and to add to the armamentarium of the current standard-of-care with the goal of impeding mesenchymal transformation in GBM.

## Biomarkers and therapeutic targets for mesenchymal glioblastoma

In addition to the master mesenchymal regulators, there are a number of genes and molecules which have been identified, through a large-scale transcriptomic analysis, as both biomarkers and therapeutic targets for mesenchymal GBM. The understanding of their interactions with each other, with master mesenchymal regulators, and also with the tumor microenvironmental factors will offer an instrumental opportunity to develop an effective and selective therapeutic modality for mesenchymal GBM patients.

As mentioned previously, one of the most well-defined biomarkers for mesenchymal GBM is genomic aberration in *NF1* locus. While EGFR and PDGFRA amplifications are the major genomic abnormalities in classical and proneural GBMs, respectively, deficiency in *NF1*, mainly via homozygous and hemizygous deletions, was observed as a highly frequent event in mesenchymal GBMs, and the pathobiological significance of such loss was associated with the infiltration of TAMs into the tumor microenvironment followed by PMT and radioresistance [11, 130, 155, 161].

Chong et al. based on the association of cell surface sialyation with tumor cell invasiveness, investigated the role of ST3GAL1 sialytransferase gene in GBM and found that this is triggered by TGFβ signaling pathway typically in mesenchymal GBM and also regulates gliomagenesis via APC/C-Cdh1-targeted control of FoxM1 protein degradation. Particularly, they showed that ST3GAL1-associated transcriptomic program favors the mesenchymal signature of GBM and is predictive of patient survival, suggesting that ST3GAL1-related processes may be a viable therapeutic target [20]. Also, S100A4, a gene that encodes a small calcium binding protein, was found as a critical upstream regulator of both EMT-associated proteins, including SNAIL2 and ZEB1, and some of the important mesenchymal signature genes in GBM, suggesting S100A4 as a critical mesenchymal marker and therapeutic target [21]. Additionally, prosaposin, which is a conserved glycoprotein with multiple biological functions, has recently been presented as a novel targetable biomarker for the treatment of mesenchymal GBM mainly because prosaposin plays a regulatory role in GBM invasion and PMT through TGFβ1/Smad signaling pathway [58]. Another critical modulator recently identified for mesenchymal GBM is sortilin, which is a member of the Vps10p sorting receptor family found to promote GBM invasion mainly via glycogen synthase kinase 3 beta (GSK-3β)/β-catenin/Twist-induced mesenchymal transition, suggesting that AF38469, a novel inhibitor of sortilin, may be a selective antitumor agent for sortilin-overexpressing mesenchymal GBM [172]. Furthermore, anti-apoptotic protein, such as BIRC3, has been identified as a biomarker for mesenchymal GBM habitats in the hypoxic microenvironment. The expression of BIRC3 was found to correlate with that of HIF-1α in a hypoxic tumor region, and it was shown that BIRC3 is a key molecule mediating the survival adaptation in hypoxia-driven mesenchymal GBM habitats [157]. Given a tight association of the mesenchymal subtype and a high degree of tumor necrosis, an enzyme such as transglutaminase 2, or TGM2, was found as a key molecular switch of necrosis-induced mesenchymal differentiation by regulating the master mesenchymal transcription factors [174].

Some microRNAs (miRNAs) have also been identified to be associated with mesenchymal GBM. miRNAs are non-coding RNAs ranging from 18 to 24 nucleotides in length that negatively regulate gene expression at the post-transcriptional level [55]. Their expression may be either contributory or inhibitory to various types of cancers, including GBM [110, 160]. One of the miRNAs found critical for mesenchymal transition in GBM is miR-23a, which was found to induce the expression of invasion- and PMT-related molecules, including RhoA, RhoC, Snail, Slug, and MMP9 [168]. On the other hand, miR-504 was found to suppress the aggressive biological processes related with the mesenchymal phenotype of GBM primarily through negatively regulating FZD7-mediated Wnt–β-catenin pathway, and, correspondingly, low miR-504/FZD7 expression ratio was found as a mesenchymal subtype marker and prognostic indicator for GBM patients [76].

The summary of the recent findings of potential targets and biomarkers for mesenchymal GBM, including those mentioned herein, is presented in Table 1. It seems that a diverse class of molecules contribute to the acquisition of the mesenchymal transcriptome in GBM. Understanding their collective involvement in the establishment of the mesenchymal signature during GBM progression will be critical to salvage patients failing multimodal therapeutic approaches applied today.

**Table 1:** A list of molecules identified to contribute to the establishment of the mesenchymal signature in GBM

Category	Name	Identified Mode of Actions	Potent Inhibitor(s)	References
Master transcriptional regulators	STAT3	Normally operate opposing signals (neurogenesis versus gliogenesis) combined expression is linked to the mesenchymal signature	YM155/AZD1480/ ruxolitinib	[16, 70, 184]
	C/EBPβ		N/A	
	TAZ	TAZ–TEAD interaction / cooperates with PDGF-B	Verteporfin	[10, 156]
Other proteins	S100A4	Regulates SNAIL2, ZEB1 and the mesenchymal signature genes	N/A	[21]
	Prosaposin	TGF-β1/Smad signaling pathway	LY2109761 (TGF‐β1 inhibitor)	[58]
	Sortilin	(GSK-3β)/β-catenin/Twist signaling axis	AF38469	[172]
	BIRC3	Induced by hypoxia	N/A	[157]
	FoxM1	Activation loop of ADAM17/EGFR/GSK3β	TAPI-2	[179]
	Nrf2	Positive feedback loop between SQSTM1/p62 and Nrf2	N/A	[118]
	PBX3	Activation of MEK/ERK1/2	N/A	[167]
	FOXO1	Regulates mesenchymal marker proteins (N-cadherin, Vimentin, CD44, and YKL‐40)	AS1842856 (type 2 diabetes mellitus)	[97]
			SIN3a (hepatic insulin sensitivity)	[68]
Enzymes	ST3GAL1	TGFβ signaling pathways	SB431542 (TGFβ inhibitor)	[20]
			AL10 (lung cancer)	[18]
	TGM2	Regulates C/EBPβ expression directly by polymerization of GADD153 via NF-κB activation	GK921	[174]
microRNAs	miR-23a	miR-23a/HOXD10 axis	N/A	[168]
	miR-10b	HOXD10/NOTCH1/TP53/PAX6 axis	N/A	[74]
	miR-504	Negatively regulates FZD7-mediated Wnt–β-catenin pathway	N/A	[76]
	miR-128a/miR-504	Negatively regulates expression of mesenchymal markers (YKL-40, CD44, and Vimentin)	N/A	[82]

## Concluding remarks

Despite much effort to characterize GBM at molecular and cellular levels, GBM still remains the most challenging solid primary tumor of the central nervous system. The large-scale genomic and transcriptomic profiling of GBMs at the various levels has provided an unprecedented knowledge of the dynamic inter- and intratumoral transcriptomic heterogeneity, which is plastic rather than static. In the present review, we have discussed molecular pathobiology associated with mesenchymal transformation in GBM and its clinical relevance; however, additional studies are required to clear several controversies over the prognosis of mesenchymal GBM patients and to target the phenotypic plasticity as an adjuvant therapy.

As evidenced by many studies, the inflammatory nature of mesenchymal GBM suggests that the immunologic status of patients, the heterogeneous activity of both immunostimulatory and immunosuppressive cell types in the tumor immune compartment, and their complex interplay with master mesenchymal regulators must be investigated to understand the impact of inflammatory microenvironment on shaping the mesenchymal signature. Such analysis may help us to develop a more effective immunotherapy for immunologically reactive, and yet, refractory mesenchymal GBMs.

Also, it has now become evident that second-line GBM therapy should consider the molecular characteristics of re-evolved tumor and consist of a targeted therapy specifically aimed at altered molecular features. To this end, a more accurate preclinical model, which effectively recapitulates the molecular characteristics of the original tumor and may be developed in a clinically relevant time frame, is urgently needed to provide a testing ground of combination of various targeted therapies.

In conclusion, GBM cells harness many different kinds of signaling pathways to their advantage to survive the therapy-insulted microenvironment; therefore, multidisciplinary therapeutic approaches, which are optimized to the unique biology of brain, should be encouraged as they may provide synergistic effects against the progressing tumor [45, 149]. In this regard, the addition of master mesenchymal regulator inhibitors may be a viable second-line therapeutic option as it may circumvent therapy-associated transcriptomic alterations in the recurrent tumor. Thus, the identification of additional master transcriptional regulators and their corresponding inhibitors may significantly improve not only the current understanding of the seemingly complex transcriptional regulatory network of mesenchymal GBM, but also the treatment response of patients with mesenchymal tumors at recurrence.

## Data Availability

Not applicable.

## Abbreviations

2-HG: 2-hydroxyglutarate
α-KG: α-ketoglutarate
CSF-1R: colony stimulating factor-1 receptor
GSK-3β: glycogen synthase kinase 3 beta
GBM: glioblastoma
GKRS: gamma knife radiosurgery
IGF-1R: insulin-like growth factor-1 receptor
LITT: laser interstitial thermal therapy
MCTs: monocarboxylate transporters
miRNAs: MicroRNAs
NF-κB: nuclear factor-κB
NF1: neurofibromin 1
PI3K: phosphatidylinositol 3-kinase
PMT: proneural-to-mesenchymal transition
EMT: epithelial-to-mesenchymal transition
TMZ: temozolomide
TCGA: the cancer genome atlas
TAMs: tumor-associated macrophages and microglia
TAZ: transcriptional coactivator with PDZ-binding motif
TEAD: transcriptional enhanced associate domain;
VEGF: vascular endothelial growth factor
